# BMP3 suppresses colon tumorigenesis via ActRIIB/SMAD2-dependent and TAK1/JNK signaling pathways

**DOI:** 10.1186/s13046-019-1435-1

**Published:** 2019-10-28

**Authors:** Jialing Wen, Xianglin Liu, Yan Qi, Feng Niu, Zhitong Niu, Wenjing Geng, Zhaowei Zou, Renli Huang, Jianping Wang, Hongzhi Zou

**Affiliations:** 1 Guangdong Institute of Gastroenterology, Guangdong Provincial Key Laboratory of Colorectal Pelvic Floor Diseases, The Sixth Affiliated Hospital, Sun Yat-sen University, Guangzhou, Guangdong China; 2 Creative Biosciences (Guangzhou) CO., Ltd., Guangzhou, Guangdong China; 3 Department of pathology, Xiaolan Hospital, Southern Medical University, Zhongshan, Guangdong China; 4 Department of General Surgery, Zhujiang Hospital, Southern Medical University, Guangzhou, China

**Keywords:** BMP3, Inhibitor, ActRIIB, SMAD2, Colorectal cancer

## Abstract

**Background:**

BMP3 gene is often found hypermethylated and hence inactivated in several types of cancers including colorectal cancer (CRC), indicating that it has a suppressor role in carcinogenesis. Though BMP3 is a reliable biomarker for screening CRC, the molecular mechanism of BMP3 in carcinogenesis remains largely unknown.

**Methods:**

The expression level of BMP3 was examined by immunohistochemistry staining and western blot. Methylation-specific PCR (MSP) and real-time quantitative MSP were used to test the hypermethylation status of BMP3 gene. Analyses of BMP3 function in colon cancer cell proliferation, migration, invasion, and apoptosis were performed using HCT116 and KM12 cells. BMP3 was further knocked down or overexpressed in CRC cells, and the effects on cell growth of xenograft tumors in nude mice were assessed. Co-immunoprecipitation and immunofluorescence staining were used to analyze the association between BMP3 and BMPR2 or BMP3 and ActRIIB. Microarray analysis was performed to identify most differentially expressed genes and pathways regulated by BMP3. The BMP3-regulated SMAD2-dependent signaling pathway and TAK1/JNK signal axes were further investigated by quantitative PCR and western blot.

**Results:**

BMP3 gene was hypermethylated and its expression was downregulated in both CRC tissues and cell lines. Expressing exogenous BMP3 in HCT116 inhibited cell growth, migration, and invasion and increased rate of apoptosis both in vitro and in vivo. However, shRNA-mediated attenuation of endogenous BMP3 in KM12 reversed such inhibitory and apoptotic effects. Furthermore, BMP3 could bind to ActRIIB, an activin type II receptor at the cellular membrane, thereby activating SMAD2-dependent pathway and TAK1/JNK signal axes to regulate downstream targets including caspase-7, p21, and SMAD4 that play crucial roles in cell cycle control and apoptosis.

**Conclusions:**

Our study reveals a previously unknown mechanism of BMP3 tumor suppression in CRC and provides a rationale for future investigation of BMP3 as a potential target for the development of novel therapeutic agents to fight CRC.

## Background

Most cases of colorectal cancer (CRC) arise from a benign to malignant transformation in cell growth, a process known as the adenoma-carcinoma sequence [1, 2], which is probably induced by the interaction between neoplastic epithelia and stromal cells through genetic or epigenetic changes in various molecular signaling pathways [3]. BMPs have profound effects on cellular development and growth. To date, studies have suggested that BMPs may play complex and multi-functional roles in tumor development and formation in various tissue types. For instance, it has been shown that BMP2 and BMP4 can act as both tumor inhibitor and enhancer in different types of cancers [4–10]. BMP6 is highly expressed in both benign and malignant human prostatic tissue [11], and its activity is suppressed by recombinant noggin, correlating with metastases of prostate cancer to skeletal bones [12]. Additionally, it has been reported that BMP7 suppresses prostate cancer growth to prevent metastasis [13]. Taken together, whether BMPs exert inhibitory or stimulating effects depends on specific tissue and cancer types. BMPs mainly bind to two kinds of type II receptors (ActRII and BMPR2) with distinct affinities, causing divergence of signaling [14] and allowing them to act as multifunctional regulators to modulate a plethora of physiological activities. BMP3, a member of the TGF-β superfamily, plays an important role in embryonic development by inducing and patterning early skeletal formation [15, 16]. It was previously reported that BMP3 could interact with activin type IIB receptor to inhibit activin signaling during embryogenesis in Xenopus embryos [17]. However, BMP3 was also shown to stimulate proliferation of human mesenchymal stem cells, a process that could be blocked by SB431542, an inhibitor of TGF-β/activin receptor kinase [18], suggesting that BMP3 could exert two-way regulatory effects on activin signaling in distinct cell types. Although it is well known that Activin, ActRII, and Alk4 (a member of TGFβ/Activin type I receptor family) coordinate to transduce signals down the activin signaling pathway [19–21], no specific receptor has been identified for BMP3 in mammals, and how interaction of BMP3 and its receptor modulates activities of its downstream effectors is still unclear. Recent studies have found that BMP3 may play a critical role in tumorigenesis in multiple types of tissues. It is found hypermethylated and inactive in gastric carcinoma [22] and cholangiocarcinoma [23]. Additionally, hypermethylation of BMP3 gene may increase the risk of breast cancer for patients with abnormal breast mass [24]. Also, BMP3 is often inactive during the early stages of most cases of CRC [25] and is hypermethylated in most colorectal neoplasms [26]. As a convenient biomarker, highly methylated BMP3 gene has been used for CRC screening in the Cologuard™ genetic test [27, 28] with high sensitivity and specificity. These findings indicate that BMP3 acts as a tumor suppressor in several types of cancers. However, the mechanism via which BMP3 activity is regulated and the role of its receptor-mediated signaling in tumorigenesis remain largely unknown, especially so in the development of CRC. In this paper, our goal is to determine what role, if any, BMP3 plays in CRC to understand why BMP3 inactivation seems to correlate with predisposition to carcinoma. First, we identified BMP3 as a potential tumor suppressor in CRC. We found that BMP3 was hypermethylated and its protein expression was significantly reduced in CRC cell lines and tissues. Then, we demonstrated that BMP3 displayed its tumor-suppressive effects in CRC both in vitro and in vivo systems. Finally, we revealed a signaling mechanism through which BMP3 ligand bound to its receptor ActRIIB to activate SMAD-dependent and SMAD-independent TAK1/JNK pathways to suppress CRC development and progression.

## Materials and methods

### Tissue samples

Samples of 37 colorectal carcinomas, 52 adenomas, and 31 normal colonic tissues, formalin-fixed and paraffin-embedded, were used for immunohistochemistry study of BMP3 expression. 80 frozen pairs of CRC samples and their matched adjacent normal colon tissues were used for performing real-time quantitative methylation-specific PCR (qMSP). Samples were obtained from the tissue bank of the Sixth Affiliated Hospital, Sun Yat-sen University (Guangzhou, China) between January 2015 and December 2017. Clinical information is summarized in Table 1. Experiments with patients’ biopsies were approved by the Institutional Review Board at the Sixth Affiliated Hospital of Sun Yat-sen University.

**Table 1:** The clinical information of samples used for qMSP analysis and immunohistochemistry

NO	qMSP analysis	Immunohistochemistry		
	Paired tissue = 80	Normal = 31	Adenoma = 52	Carcinoma = 37
Median age (range), y	60 (26–82)	59 (36–85)	56 (21–79)	61 (33–85)
Sex (M/F)	49/31	19/12	37/29	22/15
Location (proximal/distal)	19/61	20/11	30/26	21/16
TNM stage (I/II/III/IV)	20/49/7/4			12/9/7/8
Dysplasia (low/median/high/NT)	3/24/42/11			17/11/9/0

### Lentivirus and transfection

Lentiviruses were transduced into cells in the presence of 8 μg/ml polybrene (NO:141119101, Cyagen, China). After 48 h, puromycin dihydrochloride (p8833, Sigma, 2.5 μg/ml) was added to the medium for 7 days so as to select cells with stable virus integration. After drug screening, cells were collected in order to test overexpression or knockdown efficiency by real-time PCR and western blot (WB) [29]. Plasmid transfections were performed using FuGENE® HD (LOT:0000086423, Promega) according to the manufacturer’s instructions. BMP3 (NM_001201) and shRNA-BMP3 recombinant lentiviruses and their respective controls were purchased from Cyagen Bioscience Inc. (Guangzhou, China). Specific shRNA plasmids for SMAD2, TAK1, ActRIIB and BMPR2, plasmid ActRIIB-pDest-C-Myc (NM_001106), and plasmid BMPR2-pENER-C-Flag (NM_001204) were obtained from Shandong Vigene Biosciences. The shRNA sequences and the primers used are listed in Additional file 4: Table S1.

### Immunofluorescence staining

Immunofluorescence staining was performed in order to identify cellular locations of BMP3, ActRIIB, and BMPR2, using protocols as previously described [29]. For the double immunofluorescence staining of BMP3 and ActRIIB or BMP3 and BMPR2, HCT116 cells with stable BMP3 expression and wild-type KM12 cells were split in cell culture dishes with glass bottom at a proper density (30% confluence) and incubated at 37 °C for 24 h before they were fixed.

### Demethylation treatment of CRC cells

Six CRC cell lines, including HCT15, SW480, HCT116, KM12, WiDr, and DLD1, were split into 60 mm culture dishes at low density (30% confluence), grown for 24 h, and then treated with demethylation reagents 5-aza-2′-deoxycytidine (5-Aza-dC, Sigma, 5 μM/ml) and histone deacetylase inhibitor trichostatin A (TSA, Selleck Chemicals, 0.3 μM/ml), as previously reported [30, 31]. Then the total RNA was isolated using the RNeasy mini kit (Qiagen). cDNA was synthesized using the ReverTra Ace-α-kit (TOYOBO). Real Time-PCR (RT-PCR) was performed with Applied Biosystems ABI 7500. GAPDH was used as an internal control. Primer sequences are shown in Additional file 4: Table S1.

### Methylation-specific PCR (MSP)

MSP was performed in order to detect the methylation of BMP3 according to previously reported studies [32]. Briefly, after the DNA was bisulfite-modified, PCR was performed. Bisulfite-treated human genomic DNA (Novagen, Madison, WI) and CpGenome TM Universal Methylated DNA (Chemicon, Temecula, CA) were employed as positive controls for unmethylation and methylation, respectively. Methylated (M) and unmethylated (U) primers and annealing temperatures are shown in Additional file 4: Table S1. All PCR reactions were repeated three times.

### Real-time quantitative methylation-specific PCR (Q-MSP)

Fluorogenic PCRs were carried out in a reaction volume of 25 μl containing 600 nmol/L of each primer, 200 nmol/L of each probe, 5 mmol/L Mg^2+^, 400 mmol/L dNTPs, 0.1 U/mL GoTaq Hot Start Polymerase (Promega), and 1 x buffer, including 1 μL bisulfite-converted DNA. Amplifications were carried out in a Light Cycler 96. Each plate consisted of converted DNA samples, positive and negative controls, and water blank. Serial dilutions of plasmid DNA was used as standards for quantification [33]. Primers and probes are listed in Additional file 4: Table S1.

### In vivo tumor formation assay

Four weeks old male nude mice were purchased from and housed at the Experimental Animal Center, Sun Yat-sen University (Guangzhou, China). 2 × 10^6^ cells in 0.1 ml PBS were subcutaneously injected into the left axilla (10 mice per group). Each tumor was measured every 5 days for 30 days, and its volume was calculated as tumor volume = length x (width^2^)/2 as previously described [34]. Tumor images were recorded with the tumor weights assessed at the terminal time. All of the protocols for in vivo animal experiments were approved by the Ethics Committee of Sun Yat-sen University Health Science Center.

### Statistical analysis

All experiments were independently repeated at least three times. Statistical analysis was performed with SPSS version 16 or GraphPad Prism 5 software. Image analysis was used with Image J software. Data is shown as mean ± SD, with the significance between the means calculated using Two-tailed Student’s t-test. A *p* value less than 0.05 was considered statistically different (* *p* < 0.05, ** *p* < 0.01). More details see Additional file 1. Materials and Methods.

## Results

### BMP3 protein is downregulated in CRC tissues and cell lines

**Fig. 1:**
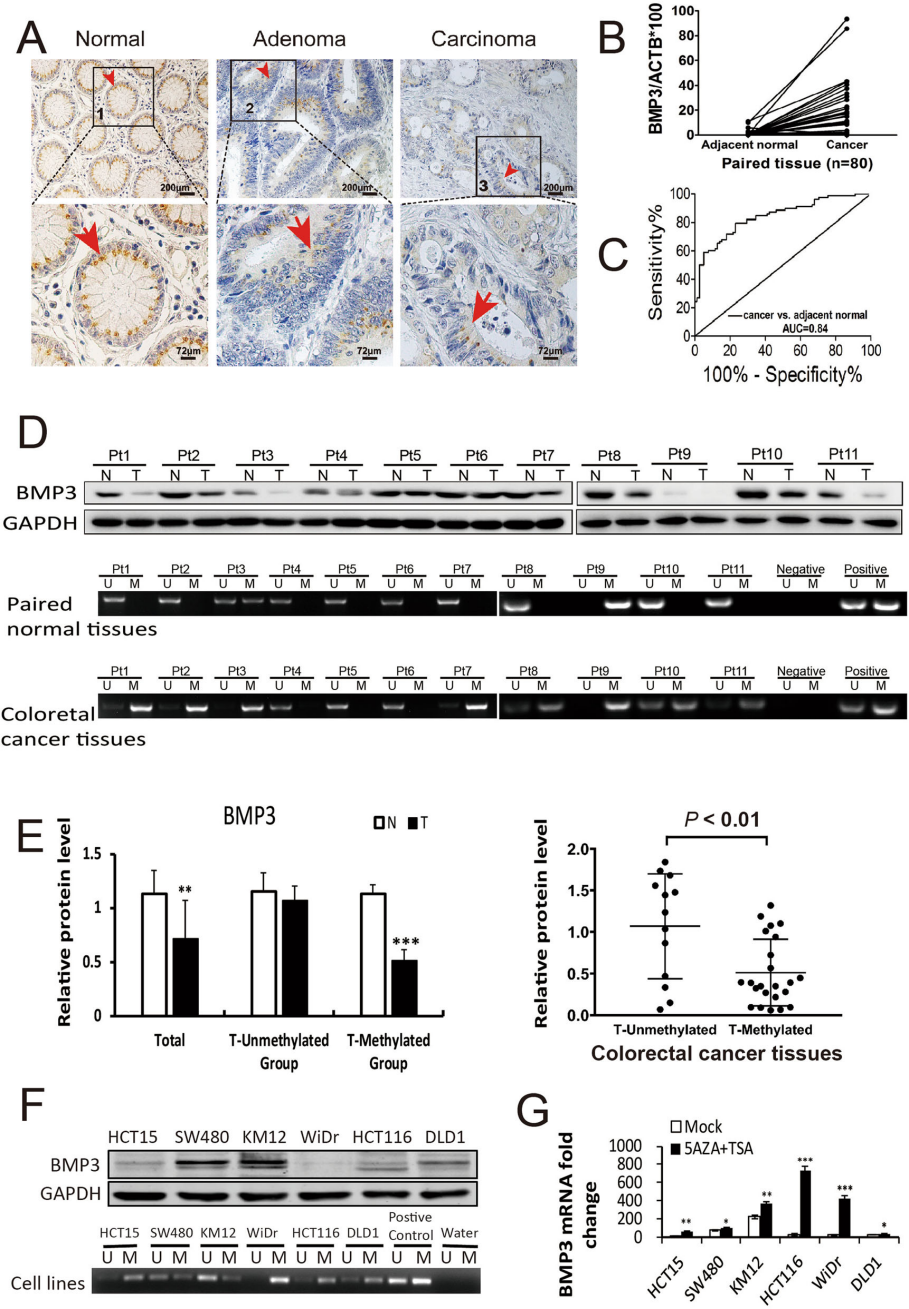
BMP3 with a hypermethylation status is downregulated in CRC. **a** BMP3 protein levels in normal, adenoma, and carcinoma tissues were assessed by immunohistochemistry (Bar, 200 μm). Enlarged images (lower panel, bar, 72 μm) and red arrows show that BMP3 is located in the cytoplasm with strong staining in normal tissue (1), moderate staining in adenoma (2), and weak staining in carcinoma. (3). **b** qMSP analysis of BMP3 methylation status in CRC tissues (Cancer) and paired adjacent normal tissues (Adjacent normal) (*n* = 80). Methylation percentages are 51.89% for cancer tissues and 5.06% for normal tissues. **c** ROC curve for BMP3 methylation levels in CRC versus paired adjacent normal tissues. **d** Upper panel: Western blot analysis of BMP3 expression in CRC tissues (T) and paired normal tissues (N). Pt: patient (*n* = 11). Middle and lower panels: Detection of BMP3 methylation status by MSP in the same paired normal and CRC tissue samples (*n* = 11), in addition to one negative control and one positive control. Lanes U and M indicate unmethylated and methylated MSP products of BMP3 gene, respectively. **e** Left panel: Relative protein expression level of BMP3 in a total of 36 sample pairs, 13 sample pairs without methylation in CRC (T-Unmethylated), and 23 sample pairs with methylation in CRC (T-methylated). Right panel: Dot histogram showing the protein expression level in 23 CRC tissues with methylation (T-methylated) and without methylation (T-unmethylated) (GraphPad Prism). BMP3 protein expression was significantly reduced in methylated group compared to that of unmethylated group (*p* < 0.01). **f** Western blot and MSP analyses in six CRC cell lines. **g** Reactivated expression of BMP3 mRNA in CRC cell lines after treatment with 5-aza-2′-deoxycytidine (5-Aza-dC) and Trichostatin (TSA) (*n* = 3). * *p* < 0.05, ** *p* < 0.01, *** *p* < 0.001

Cytoplasmic staining of BMP3 protein was significantly weaker in CRC tissues and moderately weaker in adenoma tissues than that in normal tissues (Fig. 1a). Positive staining of BMP3 protein was observed in 27 out of 31 (87.10%) normal colon epithelial specimens, 28 out of 52 (53.85%) adenoma specimens, and 13 out of 37 (35.14%) CRC specimens (Additional file 4: Table S2). WB results further demonstrated that BMP3 protein displayed lower expression level in majority of the CRC tissues (36 samples) than their paired normal counterparts (Fig. 1d, Additional file 3: Figure S1). In CRC cell lines, BMP3 protein expression was detectable at a lower level in DLD1 and HCT15 than in SW480 and KM12; however, it was hardly observed in HCT116 and WiDr (Fig. 1f).

### BMP3 gene promoter is hypermethylated in CRC tissues and cell lines

The methylation status of BMP3 was first tested in patients’ paired normal colon and CRC tissues by Q-MSP. The methylation percentage of BMP3 was 51.89% (41 of 80) in CRC and 5.06% (4 of 80) in paired adjacent normal epithelia. Natural logarithm transformed copy numbers are displayed (*p* < 0.01 for cancer vs. adjacent normal) in Fig. 1b. The area under the ROC curve (AUC) is 0.84 (95% CI, 0.878–0.90) for CRC when compared with paired adjacent normal colon (Fig. 1c), indicating strong association. MSP was further tested in 36 paired samples selected randomly from the same set of 80 paired tissue specimens to investigate the effect of methylation status on the protein expression level of BMP3. As expected, hypermethylation of BMP3 promoter resulted in a significant reduction of protein expression level of BMP3 in CRC tissues compared with that of their unmethylated paired normal counterparts (Fig. 1d, e and Additional file 3: Figure S1), showing an inverse relationship between methylation status and protein expression. In CRC cell lines, BMP3 gene promoter was heavily methylated in HCT116, HCT15, and WiDr, moderately methylated in DLD1 and SW480, and least methylated in KM12 (Fig. 1f). 5-Aza-dC treatment combined with TSA was able to upregulate BMP3 mRNA levels in the cell lines (Fig. 1g). Taken together, the hypermethylated status of BMP3 promoter is a major source of BMP3 downregulation in CRC tissues and cell lines.

### Expression of exogenous BMP3 suppresses cell proliferation in HCT116

**Fig. 2:**
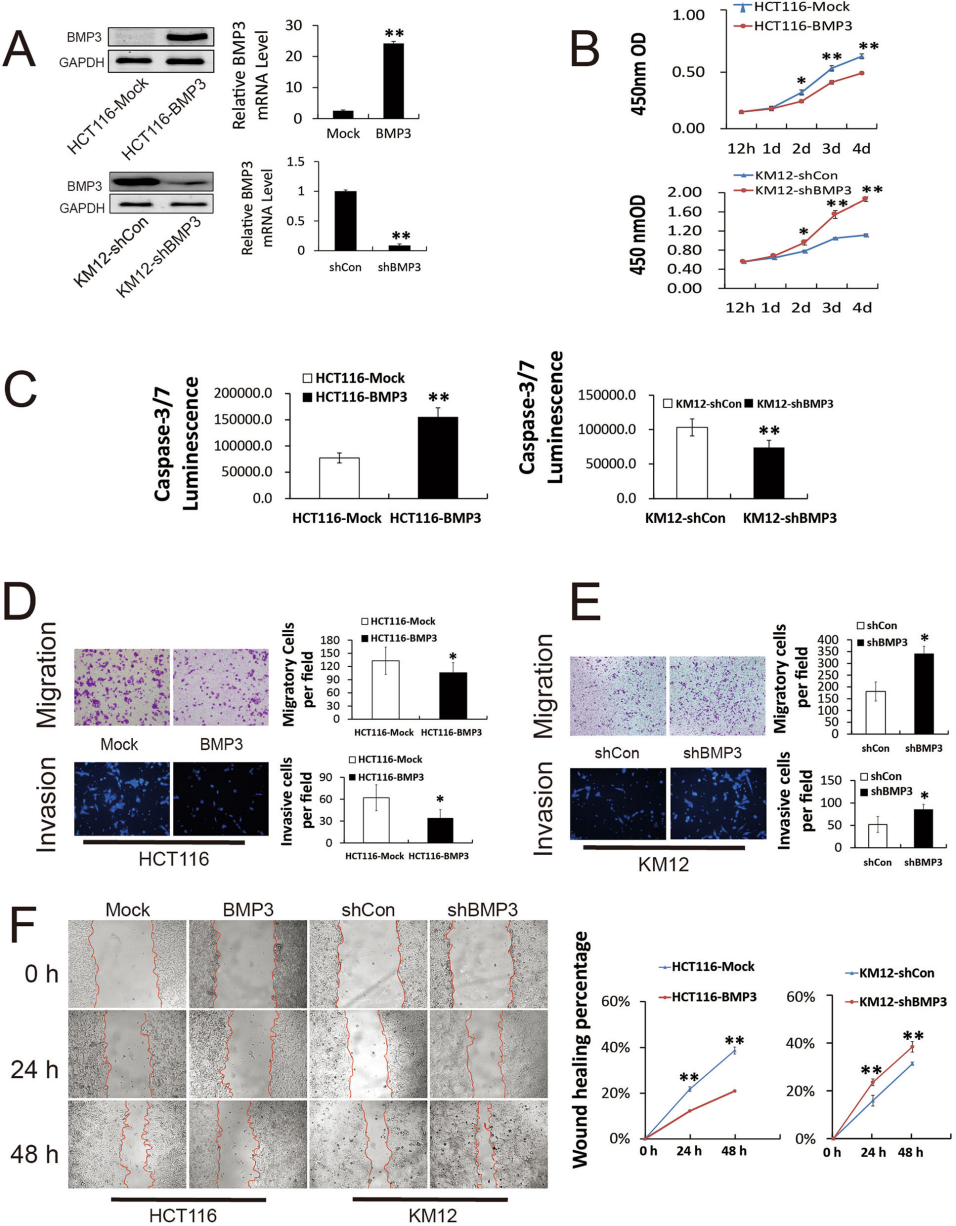
BMP3 inhibits proliferation, migration, invasion and promotes apoptosis in CRC cells. **a** qRT-PCR and western blot show BMP3 overexpression in HCT116 cells and knockdown efficiency in KM12 cells. **b** Proliferation of HCT116-BMP3 and KM12-shBMP3 cells was assessed by Cell Counting Kit-8 (CCK-8) assays. **c** Caspase-3/7 activity in HCT116-BMP3 was measured in cell lysates 24 h after cells spread. **d**, **e** Transwell migration (crystal violet staining) and invasion (DAPI staining) assays of HCT116 and KM12 cells were performed after transduction by BMP3-expression or knockdown lentivirus. **f** Wound healing assays were performed, and cells were photographed every 24 h after scratching. The wound healing percentage represents mean ± SD of at least three experiments at each time point. Statistical results were shown by bar or linear graph. * *p* < 0.05, ** *p* < 0.01

HCT116 and KM12 cells infected lentivirus carrying BMP3 coding sequence or BMP3 shRNA were used as the cell models and efficiency of infection was tested by WB and q-PCR (Fig. 2a). HCT116 cells expressing exogenous BMP3 proliferated at a much slower rate (Fig. 2b, HCT116-Mock & HCT116-BMP3). Furthermore, exogenous BMP3 enhanced activation of caspase-3/7 in the HCT116 cells (Fig. 2c) and attenuated their ability to migrate and invade in transwell assays (Fig. 2d) and wound healing experiments (Fig. 2f). In contrast, KM12 cells with reduced expression of endogenous BMP3 showed significantly enhanced activities of proliferation, migration, and invasion (Fig. 2b, e and f; shCon & shBMP3) and a decreased activity of caspase-3/7 (Fig. 2c). These data strongly imply BMP3 as a tumor suppressor in CRC development and progression.

### BMP3 inhibits tumor growth and proliferation in SCID mice

**Fig. 3:**
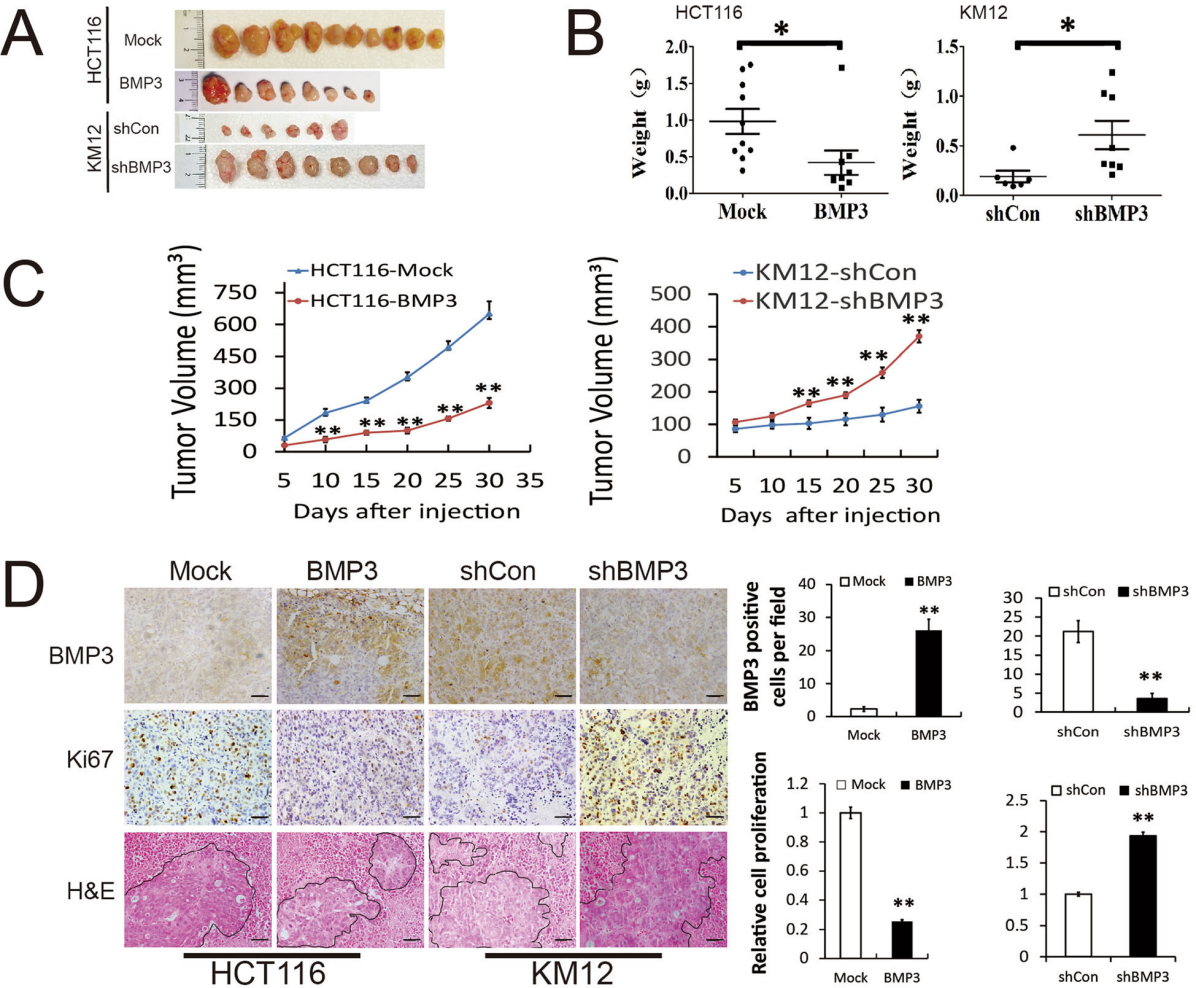
BMP3 inhibits tumor formation. **a** Effects of BMP3 stable expression and knockdown on tumor formation were evaluated in nude mice. Xenografts were collected 30 days after injection of HCT116 and KM12 cells into the armpit and photographed. **b** Each tumor was collected and its weight was assessed at the last time point. Error bars indicate SD of the mean values of each group. **c** The rate of tumor growth was measured after subcutaneous injection of HCT116 cells stably expressing BMP3 or KM12 cells with knockdown of BMP3. Data are presented as mean (tumor volume) ± SD, with *n* = 10 (Mock), *n* = 8 (BMP3), *n* = 6 (shCon), and *n* = 8 (shBMP3). T test analysis was performed to show the significant difference between the two growth curves of each group. **d** The expression of BMP3 and cell proliferation marker Ki67 was examined in xenograft tumors by immunohistochemistry. H&E staining shows the morphology of tumor cells in each group. Scale bar is 200 μm. BMP3 and Ki67 positive cells were quantified in different tumors, and data are shown as bar graphs and presented as the mean ± SD. * *p* < 0.05, ** *p* < 0.01

Modified cells were injected into the arm pits of the immunodeficient SCID mice. Localized tumors were collected 30 days after injection, and their weights and volumes were analyzed as shown in Fig. 3a. Significant growth reduction in HCT116-BMP3 tumors was observed compared to the control group, showing statistically significant differences in tumor weights and volumes between these two groups. In contrast, drastic increases in weights and volumes of KM12-BMP3-shRNA tumors were observed compared to their matching controls, exhibiting again the tumor-enhancing effects of BMP3 knockdown (Fig. 3b and c). Further histological examination of the tumor xenografts from SCID mice revealed a much higher degree of BMP3 expression in HCT116-BMP3 tumor cells, resulting in decreased Ki67 staining (Fig. 3d). The cell mass of the HCT116-BMP3 tumor xenografts shrank significantly, while enlarged nuclei were observed in a large proportion of the KM12-BMP3-shRNA tumor cells by H&E staining (Fig. 3d). From these results, we can conclude that BMP3 can strongly inhibit tumor formation and growth in vivo.

### BMP3 forms a complex with a type II receptor ActRIIB at CRC cell membrane

**Fig. 4:**
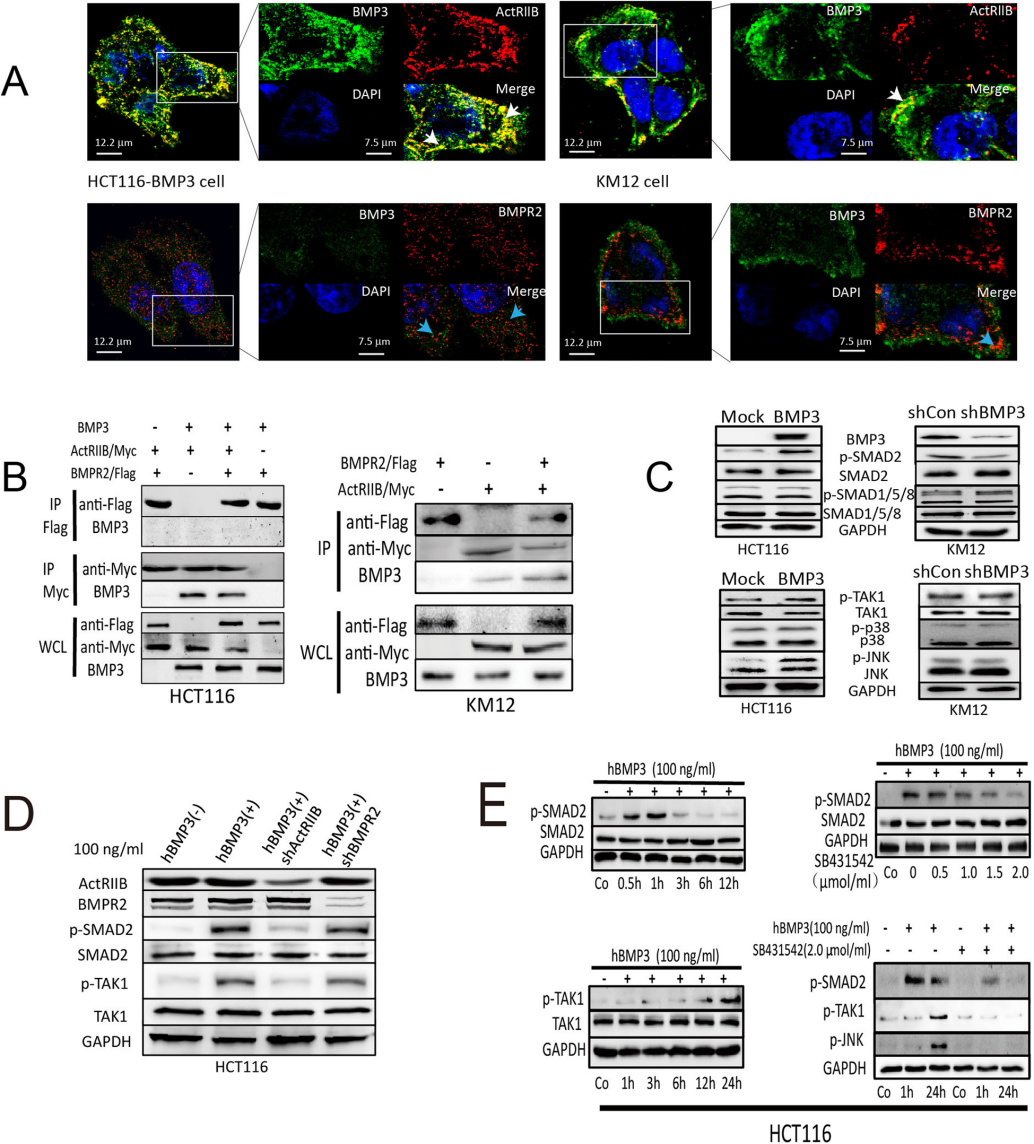
Binding of BMP3 to ActRIIB activates SMAD2, TAK1, and JNK, which is blocked by specific inhibitor SB431542. **a** Co-localization of BMP3 with ActRIIB, not BMPR2, was observed in HCT116-BMP3 cells. Enlarged images show co-localization (yellow and white arrows) of BMP3 (green) with ActRIIB (red). No co-localization of BMP3 (green) with BMPR2 (red and blue arrows) was observed (no yellow and blue arrows). White arrows indicate co-localization of BMP3 and ActRIIB and blue arrows indicate BMPR2 membrane receptor. White bar in original image is 12.2 μm and in enlarged image is 7.5 μm. **b** BMP3 was co-expressed with ActRIIB or BMPR2 in HCT116 cells, which were incubated for 48 h before they were harvested. Cell lysates were precipitated with anti-Flag antibody or anti-Myc antibody to detect the interaction of BMP3 with ActRIIB or BMPR2 by western blot. KM12 cells were also transfected with constructs carrying ActRIIB alone, BMPR2 alone, or both to detect which membrane receptor binds to endogenous BMP3. **c** Overexpression of BMP3 in HCT116 cells up-regulates the phosphorylation levels of SMAD2, TAK1, and JNK, but not SMAD1/5/8 or p38 (left panel). BMP3 knockdown reversed the up-regulation effects of p-SMAD2, not p-TAk1 or p-JNK in KM12 cells (right panel). **d** Knockdown of ActRIIB, rather than that of BMPR2, significantly reduced p-SMAD2 and p-TAK1 in the presence of BMP3. **e** Peak levels of p-SMAD2 and p-TAK1 were reached at 1 h and 24 h, respectively, after treatment of HCT116 cells with recombinant human BMP3 at 100 ng/ml. SB431542 invalidated the phosphorylation effects induced by hBMP3 in a concentration dependent manner. The activation of TAK1 was inhibited by SB431542 at 24 h time point

To determine which receptor BMP3 binds to, double immunofluorescence staining (IF) was performed for BMP3 and ActRIIB or BMPR2. We found that BMP3 co-localized with ActRIIB at the plasma membrane (the white arrow heads in Fig. 4a) of HCT116-BMP3 cells. However, no co-localization of BMP3 with BMPR2 was observed in the same cells. The co-localization of endogenous BMP3 with ActRIIB, but not BMPR2, was further observed in KM12 at the cell surface, suggesting that this ligand-receptor interaction is specific. Association of BMP3 and ActRIIB was further demonstrated by the co-immunoprecipitation (co-IP) assay. Epitope antibodies against Myc-tagged ActRIIB could precipitate both exogenous and endogenous BMP3 protein in HCT116-BMP3 and KM12 cells; however, anti-Flag antibodies could not pull down BMP3 with Flag-tagged BMPR2 in either cell lines (Fig. 4b). These results suggest that BMP3 may bind to ActRIIB at the CRC cell surface to initiate its signaling.

### BMP3-ActRIIB complex activates SMAD2-dependent and TAK1/JNK pathways

Next, we investigated the downstream targets as a result of binding of BMP3 and ActRIIB. Compared to the scrambled controls, WB analysis showed that the expressing exogenous BMP3 elevated p-SMAD2, p-TAK1, as well as its downstream molecule p-JNK, but not p-p38 (Fig. 4c). In contrast, BMP3 knockdown resulted in the downregulation of p-SMAD2, but it had no effect on p-TAK1 and p-JNK levels in KM12 cells. Additionally, no change was observed for the phosphorylation levels of SMAD1/5/8 as a result of BMP3 overexpression or attenuation (Fig. 4c). These data suggest that exogenous BMP3 can activate both SMAD2-dependent and independent pathways. However, the knockdown of endogenous BMP3 attenuated the SMAD2-dependent pathway only, significantly reducing p-SMAD2 rather than perturbing TAK1/JNK axis. Furthermore, when ActRIIB was downregulated in HCT116-BMP3 cells, we observed a drastic reduction in p-SMAD2 and p-TAK1 (Fig. 4d), implying that BMP3 activates its downstream signaling pathways through targeting its type II receptor ActRIIB. Next, we found that SB431542 was the most effective inhibitor of p-SMAD2, while other drugs, such as DMH1 and ML347, resulted in noticeable reduction of basal phosphorylation level of SMAD1/5/8 (Additional file 3: Figure S2A). Subsequently, our study showed that the peak SMAD2 activity occurred just 1 h after treatment of human recombinant BMP3 (hBMP3); however, it was delayed until 24 h later for TAK1, which was inhibited by SB431542 in a concentration-dependent manner (Fig. 4e). The inhibitory effect of SB431542 was further tested on both HCT116-BMP3 and WiDr-BMP3 cells, in which upregulation of p-SMAD2, p-TAK1, as well as p-JNK was observed. When SB431542 was added to the cultured cells, p-SMAD2 was drastically reduced after 1 h treatment and was hardly detectable by 24 h. In contrast, the phosphorylation level of both TAK1 and JNK remained unchanged after 1 h and was significantly reduced after 24 h (Additional file 3: Figure S2B). These data further support the notion that the BMP3-ActRIIB complex activates SMAD2-dependent signaling pathways as well as the TAK1/JNK signal axis, evidenced by immediate upregulation of p-SMAD2 and delayed activation of TAK1 and JNK.

### Expression profiling reveals dysregulation of biological pathways in HCT116-BMP3 cells

**Fig. 5:**
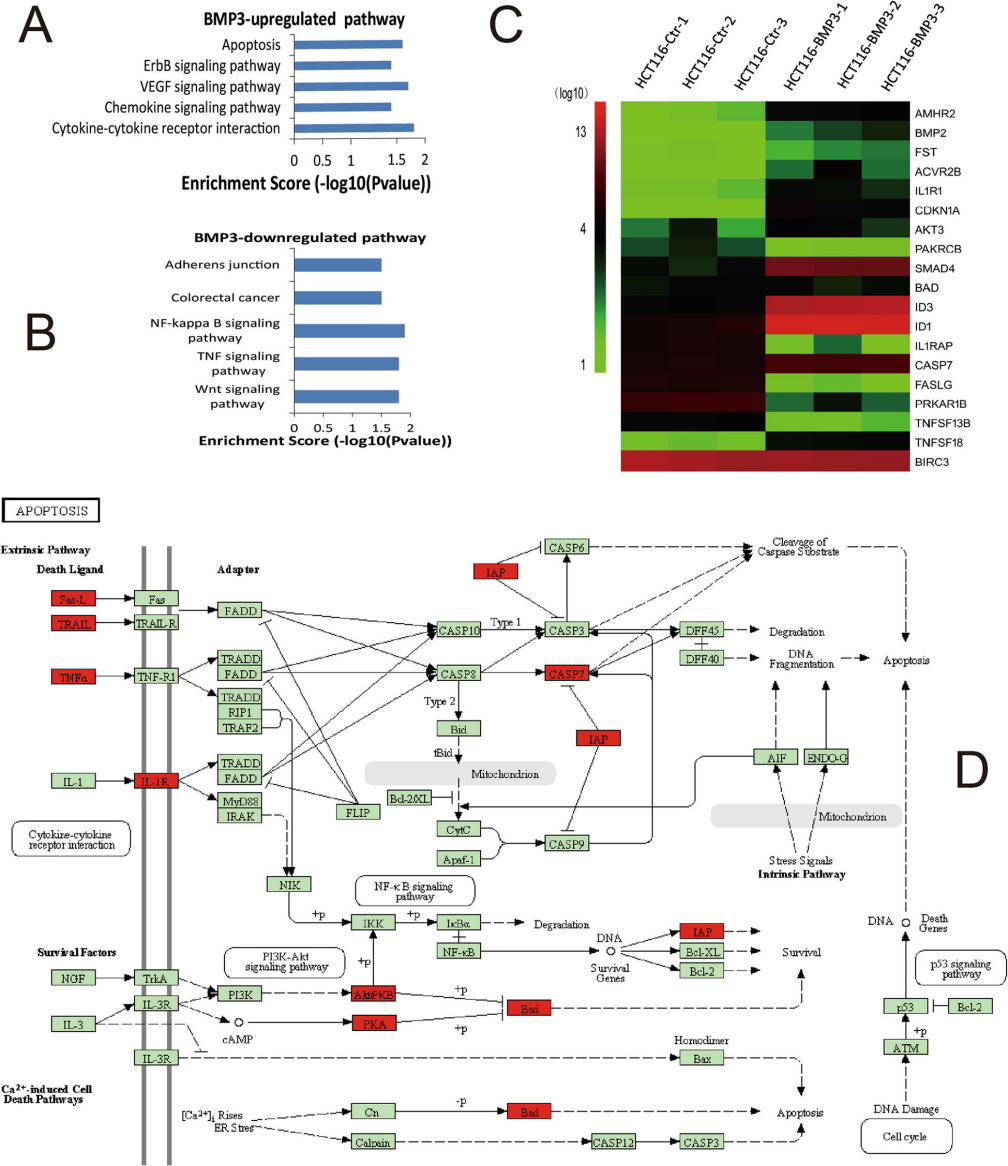
BMP3 regulates multiple cellular processes in HCT116 cells. Global mRNA expression profiling was performed for HCT116-BMP3 and control cells (HCT116-Ctr). The experiment was repeated three times for each cell type. KEGG pathway analysis was carried out and affected pathways were categorized as up-regulation (**a**) and down-regulation (**b**). **c** A heat map was generated from hierarchical cluster analysis to visualize genes with differentially expressed mRNAs in the apoptosis and TGF-β signaling pathways. Red and green colors represent those genes with either high or low relative expression, respectively. **d** Components of apoptosis pathways are displayed. Genes highlighted red are up-regulated. KEGG is Kyoto Encyclopedia of Genes and Genomes

Geneontology (GO) analysis shows that genes regulated by BMP3 can be classified into three main categories: biological processes, molecular functions, and structural components (Additional file 3: Figure S3A, B, and C), and they are involved in a variety of cellular functions. To elucidate the mechanism underlying growth inhibition by exogenous BMP3, we performed KEGG pathway analysis and found that genes in apoptotic pathways were mostly upregulated in HCT116-BMP3 cells compared to control cells (Fig. 5a). In contrast, genes in TNF and Wnt signaling pathways were attenuated (Fig. 5b). Genes modulated by exogenous BMP3 as identified in the afore-mentioned GO and KEGG pathway analyses are all displayed in the Additional file 2. Additionally, we performed microarray analysis of transcriptional profile of HCT116-BMP3 cells and obtained a heat map displaying the genes that are most affected by BMP3 in TGF-β signaling and apoptosis pathways, namely AMHR2, BMP2, FST, ACVR2B, IL1R1, CDKN1A, et al. (Fig. 5c). Illustration of the apoptotic pathways is presented with upregulated genes highlighted red in the pathways (Fig. 5d). Genes of several important signal transduction proteins CDKN1A, SMAD4, and CASP7, functioning in cell cycle regulation, TGF-β signal transmission, and apoptosis control, were enriched by the overexpression of BMP3 in HCT116 cells. These data further support the notion that BMP3 inhibits the growth of CRC cells by regulating multiple vital biological processes.

### Validation of selected genes identifies SMAD4, caspase-7, and p21 as critical downstream targets

**Fig. 6:**
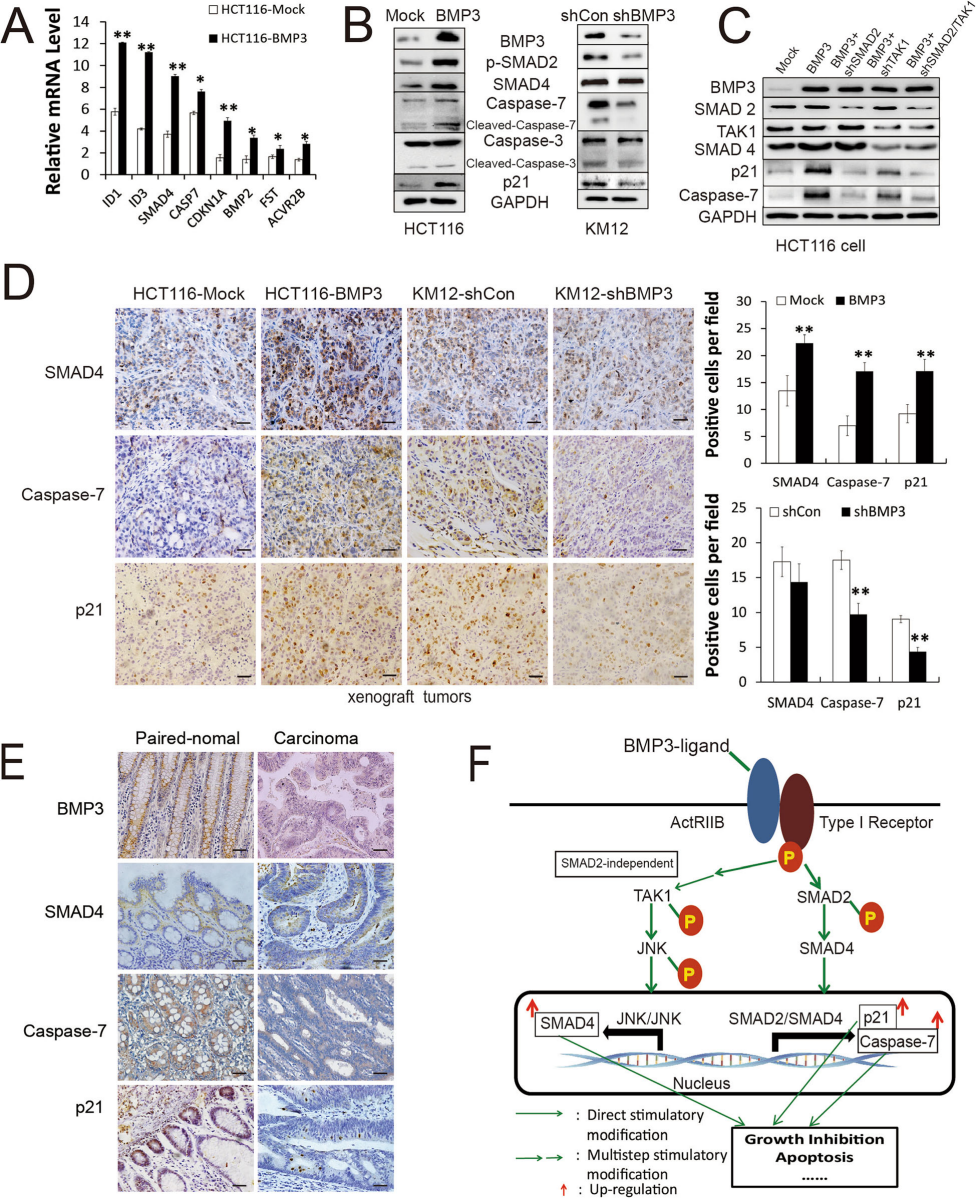
BMP3 upregulates SMAD4, p21, and caspase-7 expression. **a** Quantitative RT-PCR was used to validate the expression levels of selected genes from the microarray data in HCT116-BMP3 cells. Data are presented as mean ± SD, *n* = 3. **b** WB analysis of SMAD4, p21 and caspase-7 were performed for HCT116-BMP3 and KM12-BMP3-shRNA cells. **c** The expression levels of SMAD4, p21, and caspase-7 were measured after knockdown of SMAD2, TAK1, or SMAD2/TAK1 via SMAD2 and TAK1 shRNA interference. **d** The expression of SMAD4, p21, and caspase-7 were examined by immunohistochemistry in xenograft tumors with BMP3 overexpression or knockdown. Scale bar is 200 μm. Bar graphs show the quantified results of cells stained positive for SMAD4, caspase-7, and p21 in xenograft tumors. Data were presented as the mean ± SD. **e** A correlation between the expression of BMP3 and SMAD4, p21, and caspase-7 was found in paired-normal and carcinoma tissues. Scale bar is 200 μm. **f** Schematic diagram of SMAD2-dependent and SMAD2-independent signaling pathways regulated by BMP3 in CRC. Red arrow, up-regulation; P, phosphorylation. * *p* < 0.05, ** *p* < 0.01

The expression level of a panel of selected genes including ID1, ID3, SMAD4, CASP7, CDKN1A, BMP2, FST, and ACVR2B was further validated by qRT-PCR. Expression of exogenous BMP3 elevated mRNA levels of these genes (Fig. 6a). WB analysis of SMAD4, caspase-7, and p21 showed that these proteins were upregulated, which is consistent with their rising mRNA levels in HCT116-BMP3 cells compared to the control ones. In contrast, protein levels of caspase-7 and p21 were decreased as a result of knockdown of BMP3 in KM12 cells, while that of SMAD4 was not affected (Fig. 6b). Even though we observed increased activity of caspase-3/7 assays in HCT116-BMP3 cells (Fig. 2c), we found that, unlike caspase-7, caspase-3 expression level remained unchanged in both HCT116 and KM12 cells (Fig. 6b). Since both SMAD2-dependent and TAK1/JNK pathways are activated, we performed the following studies to elucidate which one targets these downstream effectors of SMAD4, caspase-7, and p21 when BMP3 is expressed. As shown in Fig. 6c, the expression of p21 and caspase-7 was drastically reduced as a result of SMAD2 and TAK1 knockdown, while TAK1 ablation resulted in the reduction of SMAD4. Additionally, HCT116-BMP3 cells had higher protein levels of SMAD4, p21, and caspase-7 compared to the mock group. The absence of BMP3 led to the reduction of p21 and caspase-7 but had little effect on SMAD4 in KM12 cells (Figs. 6d). Moreover, similar results were observed in paired CRC tissues and tumor xenografts by IHC (Fig. 6e, Additional file 3: Figure S4). These data indicate that BMP3 can modulate caspase-7 and p21 expression via SMAD2/SMAD4 signaling pathway, while knockdown of TAK1 can reduce SMAD4 protein even with BMP3 expression. Taken all together, our results strongly support the notion that activation of both SMAD2-dependent pathway and TAK1/JNK signaling axis, which regulate crucial downstream targets in cell cycle control and apoptotic regulation, is essential for BMP3’s tumor suppressor role in CRC development and progression (Fig. 6f).

## Discussion

In the present study, we found that BMP3 gene was extensively hypermethylated and downregulated in CRCs. The results are consistent with previous reports that BMP3 silencing is an early and frequent event in CRC progression [25]. Similarly, our results showed that BMP3 expression was reduced when its gene promoter was hypermethylated in colorectal tumor tissues in addition to colon cancer cell lines. Moreover, treatment with 5-Aza-dC and TSA could restore BMP3 expression in cultured cell lines. Altogether, hypermethylation of BMP3 gene promoter accounts as a primary source for the loss of BMP3 expression and activity in CRCs. As a result, the methylation pattern of BMP3 gene promoter regions requires further exploration.We observed broad inhibitory effects of BMP3 on cell growth, migration, and invasion, accompanied by increasing rate of apoptosis in our cultured cell models, and such effects were also observed in an in vivo system of SCID mice. Similarly, previous reports showed that BMP3 could significantly reduce colony formation in CRC cell lines [25] and suppress growth activity in biliary cancer cells [23]. Collectively, these observations support the hypothesis that BMP3 acts as a tumor suppressor or has a growth rate-limiting role in CRC development and progression.We further demonstrated that BMP3 binds to activin type IIB receptor in HCT116 and KM12 cells by IF staining and IP assays, which is consistent with previous reports [17, 18]. We have shown that BMP3 initiates TGF-β/Activin signaling by forming a complex with ActRIIB and hence activates SMAD2-dependent pathways and TAK1/JNK signal axes, suppressing growth and proliferating activities in CRC cells. It is interesting to note that BMP3/ActRIIB/ALK4 inhibits the activity of SMAD2 to negatively modulate embryogenesis in Xenopus [17], but BMP3 increases p-SMAD2 to promote proliferation through the same signaling channel in a mesenchymal stem cell (C3H10T1/2cells) [18]. These data suggest that BMP3 may display distinguishable functions via modulation of its signaling pathways of ActRIIB, ALK4, and SMADs. This functional variation mainly arises from cell-context dependence of BMP proteins. In fact, BMP3 had no effect on ERK1/2, p38, or JNK in C3H10T1/2 cells [18], whereas it increased the activity of TAK1/JNK in HCT116 cells. These data suggest that different sets of genes targeted by BMP3-ActRIIB complex determine which pathways, SMAD-dependent or TAK1/JNK signal axes, will be activated and hence the fate of each specific type of cells.A considerable basal level of p-SMAD1/5/8 remained unchanged when BMP3 was overexpressed or knocked down. DMH1 and ML347, selective inhibitors of activin type I receptors (ALK2) [35, 36], significantly reduced p-SMAD1/5/8. This further supports the notion that BMP3 activates ActRIIB/ALK4 but not BMPR2/ALK2 pathways. The peak phosphorylation of SMAD2 in HCT116 appeared only 1 hour after treatment of recombinant BMP3 while that of TAK1 was delayed by 24 h, indicating that TAK1 might be activated as a result of p-SMAD2. To test this hypothesis, we used SB431542, a potent selective inhibitor of SMAD2, to treat the cultured HCT116 cells. As predicted, SB431542 drastically decreased p-SMAD2 in the presence of recombinant BMP3, leading to significant reduction of both p-TAK1 and p-JNK. When HCT116-BMP3 and WiDr-BMP3 cells were treated with SB431542, similar phosphorylation patterns of SMAD2 and TAK1 were also observed. These data indicate that the activation of TAK1/JNK is regulated by BMP3/SMAD2 signaling pathway in cells with high level expression of BMP3. The immediate activation of SMAD2-dependent signaling and a subsequent delayed TAK1/JNK response, are mainly responsible for the cell growth retardation, migration inhibition, invasion suppression, and facilitation of cell apoptosis observed in HCT116 cells.RNA profiling was used to analyze the molecular signature of HCT116-BMP3 cells, revealing significant mRNA expression changes in multiple signaling pathways. This profile revealed that the mRNA levels of several genes, which negatively regulate cellular activities, were upregulated in the presence of BMP3. One of them was ID3, which was shown to induce cell growth arrest and caspase-3-dependent apoptosis [37, 38]. Though expression level of caspase-3 mRNA was not altered, we did observe an upregulation and activation of caspase-7 induced by BMP3, which likely led to increased rate of apoptosis. We speculate that the elevation of caspase-7 mRNA is due to increased expression level of ID3, and this hypothesis needs to be investigated in our future studies. Two more crucial genes, BMP2, a member of the TGFβ superfamily, and SMAD4, the central factor of TGFβ signaling pathways, were also upregulated. Both genes have been previously shown to inhibit CRCs [17, 39, 40] and may play tumor suppressor roles in HCT116 cells with stably expressed BMP3. Another interesting gene that we have found upregulated is FST, an intrinsic inhibitor of activin [41]; its encoded protein, together with ACVR2B (known as ActRIIB), may inhibit cancer cell growth via antagonizing activin signaling in our cell model. In addition, the enhanced mRNA level of cell-cycle gene CDKN1A (known as p21) may prevent cell proliferation, which is consistent with previous findings. Several studies have revealed an association between p21 downregulation and metastasis, as well as poor survival, in CRC patients [42–46]. Since caspase-7, p21, and SMAD4 play crucial roles in cell cycle control and apoptosis, we further investigated how these proteins were regulated in HCT116-BMP3 and KM12 cells. One thing worth noting is that, unlike caspase-7 and p21, SMAD4 was not downregulated by BMP3 knockdown in KM12 cells, which was similar to TAK1 activation in the same cells. It was reported that the total p21 protein expression could be either dependent or independent of SMAD4, according to the status of activin or TGFβ [47]. It was also found that both SMAD2 and TAK1 silencing could downregulate expression of caspase-7 and p21. Moreover, TAK1 silencing also significantly reduced the protein level of SMAD4. It appears, then, that TAK1/JNK/SMAD4 form a positive feedback loop to maintain and enhance the signals that BMP3 initiated.

## Conclusion

In conclusion, our study reveals a previously unknown mechanism of BMP3 tumor suppression in CRC. The present data illustrates a scheme in which a signal is initiated by BMP3, propagated through p-SMAD2 and p-TAK1/p-JNK, and finally transduced to downstream effectors including caspase-7, p21, and SMAD4 to induce apoptosis and inhibit CRC cell growth, migration, and invasion. Our investigation provides evidence to support the notion that BMP3 is potentially a rational target for developing novel therapeutic agents against CRC.

## Supplementary information

**Additional file 1.** Materials and Methods.

**Additional file 2.** GO and KEGG pathway analysis.

**Additional file 3:** **Figure S1.** (A) Western blot and MSP analyses of BMP3 in additional CRC tissues and their paired normals (*n* = 25). N: Paired normal tissue, T: CRC tissue, U: Unmethylated, M: Methylated. (B) Correlation between the methylation level of BMP3 promoter and the relative BMP3 protein level of 23 methylated CRC samples by linear regression. **Figure S2.** (A) Cells were treated for 1 h (1 h) with or without 2 μmol/ml of DMH1, SB431542, SB525334, or ML347 in serum-free medium, followed with 100 ng/ml hBMP3 for another 1 h. Whole cell lysates were then subjected to western blot analysis using antibodies against p-SMAD2, p-TAK1, and p-JNK. (B) SB431542 was added to the cultured cells of HCT116-BMP3 and WiDr-BMP3 to detect the effects of the inhibitor on p-SMAD2, p-TAK1, and p-JNK by western blot. All experiments were repeated at least three times. Co: control. **Figure S3.** Geneontology (GO) enrichment analysis was performed in 3 categories (*p* < 0.05): (A) biological processes, (B) molecular functions, and (C) cellular components. **Figure S4.** Synchronized expression of caspase-7 and p21 in SCID mice xenograft tumors. BMP3 was stained brown in kytoplasm; caspase-7 was stained red in kytoplasm, and p21 was stained brown in nucleus. The scale bar is 40 μm.

**Additional file 4: Table S1.** shRNA sequences and Primers for PCR. **Table S2.** Immunohistochemistry showed the expression of BMP3 in tissue samples.

## Data Availability

All data generated or analyzed during this study are included in this published article.

## Abbreviations

ActRIIB: Activin Receptor II B
BMP2: Bone Morphogenetic Protein 2
BMP3: Bone Morphogenetic Protein 3
BMP4: Bone Morphogenetic Protein 4
BMP6: Bone Morphogenetic Protein 6
BMP7: Bone Morphogenetic Protein 7
BMPR2: Bone Morphogenetic Protein receptor 2
BMPs: Bone Morphogenetic Proteins
CRC: Colorectal cancer
JNK: c-Jun N-terminal kinase
p-SMAD2: phosphorylation-SMAD2
SCID: Severe Combined Immunodeficiency
shRNA: short hairpin RNA
SMAD2: Drosophila Mothers Against Decapentaplegic Protein 2
TAK1: Transforming growth factor activated kinase-1
