# Supplementary material for: BMP3 suppresses colon tumorigenesis via ActRIIB/SMAD2-dependent and TAK1/JNK signaling pathways
Source: J Exp Clin Cancer Res. 2019 Oct 28;38:428. doi: 10.1186/s13046-019-1435-1 (PMC6819484; doi:10.1186/s13046-019-1435-1)
Supplement: Supplementary file 1 — Additional file 1. Materials and Methods. [file 13046_2019_1435_MOESM1_ESM.docx]

**Additional file 1: Materials and Methods**

**Cell lines and cell culture**

Human colon carcinoma cell lines, HCT-15, WiDr, SW480, HCT116, and DLD1, were obtained from the American Type Culture Collection (ATCC). KM12 was purchased from the Japanese Collection of Research Bioresources (JCRB). HCT-15, KM12, HCT116, and DLD1 were maintained in RPMI 1640 (Gibco), SW480 was cultured in Leibovitz's L-15 (Gibco), and WiDr was maintained in DMEM (Gibco), with all of them supplemented with 10% fetal bovine serum (Gibco) and 100mg/l Penicillin/streptomycin. All cells were grown in monolayers in a humidified atmosphere containing 5% CO_2_ at 37°C. Cells were authenticated by short tandem repeat (STR) DNA profiling by VivaCell Biosciences (Shanghai, China), Beijing Microread Genetics (Beijing, China), and Guangzhou Jennio Biotechnoligy (Guangzhou, China), and routinely tested for mycoplasma contamination with a MycoGuard™ Mycoplasma Bioluminescent Detection Kit (Fulengen, China).

**Immunohistochemistry and H&E staining**

Sections of normal colon tissue, adenoma tissue, colorectal cancer tissue, and xenograft tumor tissue samples were used for immunohistochemistry (IHC). The procedure was conducted as previously reported [1]. The commercially available antibodies *BMP3* (ab134724), anti-*ki-67*(DIA-670-P05), anti-*Smad4* (CST-46535), anti-c*aspase-7* (CST-9492), and anti-*p21* (ab109520) in 1:100 dilution were used to stain sections. The intensity of the specific immunohistochemical staining reactions were evaluated using a semi-quantitative method (IRS-score), as previously described [2]. H&E staining was carried out using a Hematoxylin and Eosin Staining Kit (Beyotime, Shanghai, China) according to the manufacturer’s protocol.

**Western blot and immunoprecipitation**

Experiments were performed according to the protocol previously described [3]. 30 μg of total protein extraction was resolved by SDS-PAGE and transferred to nitrocellulose membranes. Samples were incubated overnight at 4°C with primary antibodies. Then, the proteins were incubated with Goat anti-Mouse IgG or Goat anti-Rabbit IgG secondary antibodies and detected using a ChemiDoc Imaging System (BIO-RAD). All antibodies were diluted to 1:1000, except for anti-Phospho-Smad1 and anti-Phospho-*Smad2* (1: 500). The anti-*GAPDH* (1: 5000) antibody was used as a loading control.

The following antibodies were used: anti-*BMP3* (ab134724), anti-*BMPR2* (ab106266), anti-*ActRIIB* (ab76940), anti-*p38* (ab31828), anti-*p21* (ab109520), anti-*JNK1* (ab199380), anti-Phospho*-JNK1* (ab215208), anti-Phospho-*p38* (ab4822), anti-*Smad2* (CST-3122), anti-Phospho-*Smad2* (CST-3104), anti-*Caspase3* (CST-9662), anti-*Smad4* (CST-46535), anti-Phospho-*TAK1* (CST-9339S), anti-*Caspase7* (CST-9492), anti-*Smad1* (CST-9743), anti-Phospho-*Smad1* (CST-5753), anti-*TAK1* (CST-D94D7), anti-*myc-*Tag (CST-2276), goat-anti-Mouse-IgG (A0216, Beyotime), goat-anti-Rabbit-IgG (A0208, Beyotime), anti-*GAPDH* (60004-1-Ig, ProteinTech), DAPI (D21490, Invitrogen), and anti-flag (F2555, Sigma).

Lysis buffer for immunoprecipitation (IP) was prepared according to previous reports [4]. 400 μg cell lysate was incubated with 2 μg of antibodies and 50 μg of Protein A/G PLUS-Agarose (sc-2003) overnight at 4°C. Beads were washed four times with lysis buffer and then loaded onto PAGE gels for western blotting (WB) .

**Pictures acquired and antibodies used for immunofluorescence staining**

Fluorescence confocal images were captured by a Laica TCS SP8 using either a ×40 or ×20 lens and Laser Scanning Microscope Leica LAS AF Lite software. Primary antibodies anti-BMPR2 (ab96826), anti-BMP3-C-20 (sc-7404), and anti-ActRIIB (NBP2-58902) were incubated at 4°C overnight, with Donkey-anti-Goat (Alexa Fluor 488, 705-545-147, Jackson), Goat-anti-Mouse (Alexa Fluor plus 555, A32727, Invitrogen), and Goat-anti-Rabbit (Alexa Fluor plus 594, A32740, Invitrogen) used for secondary detection.

**Microdissection and DNA extraction**

A pathologist re-examined the tissue sections and circled out histologically distinct lesions, including adenocarcinoma with different grades and normal epithelia. Ten-micron frozen tissue sections with histologically distinct lesions were carefully microdissected. DNA of tissues and cell lines were extracted using Qiagen DNA Mini Kit (Qiagen, Valencia, CA).

**Bisulfite treatment**

Genomic DNA extracted from cell lines and tissue samples was bisulfite treated using EZ DNA Methylation Kit (Zymo Research) according to the manufacturer's instructions. Details were carried out as previously described [5]. 2 μg of genomic DNA was treated with sodium bisulfite in each reaction and diluted out in 30 μl of TE buffer.

**Real-time quantitative methylation-specific PCR (Q-MSP)**

The bisulfite-modified DNA samples from CRC tissue and paired normal tissue were used as a template for fluorescence-based real-time PCR (Taqman), as previously described [6]. Primers and probes were designed in order to amplify the bisulfite-converted promoter of *BMP3* and *ACTB* that is used as an internal reference gene. The methylation level of the BMP3 gene was defined as the ratio (the copies of *BMP3*/that of *ACTB*) and multiplied by 100 to give a percentage value [2].

**Procedures for methylation-specific PCR (MSP) and real-time quantitative methylation-pecific PCR (s-MSP)**

**MSP**

The amplification included a hot-start at 95°C for 12 min, denaturing at 95°C for 30 sec, annealing at 59°C for 60 sec, extension at 72°C for 30 sec for 40 cycles and a final 10 min extension step.

**Q-MSP**

Cycling conditions followed 95℃ for 5 minutes, 10 cycles at 95℃ for 30 seconds, 65℃ for 60 seconds, and 72℃ for 30 seconds; 35 cycles at 95℃ for 30 seconds, 60℃ for 60 seconds, and 72℃ for 30 seconds; and a final cooling step at 37℃ for 30 seconds.

**Cell proliferation assay**

Measurements of cell proliferation used a Cell Counting Kit-8 (CCK-8) assay kit (Dojindo, Kumamoto, Japan). 2,000 cells were planted into each well of a 96-well plate, in which 10 μL CCK-8 was added to 90 μl of the culture medium. After incubated for 2 h at 37℃, the cells were measured at 450 nm using a Thermo Fisher Multiskan at time points of 12 h, 1 d, 2 d, 3 d, and 4 d. Three independent experiments were performed.

**Migration and Invasion Assays**

*In vitro* migration assays of 500 μl cell suspension (4×10^4^ cells) were placed into the top chamber of each insert (8.0 μm, Costar, Cambridge, MA, USA). For the invasion assay, 8×10^4^ cells were added to the upper chamber of each insert and coated with 50 μg Matrigel (BD Biosciences). After 24 h incubation at 37°C, cells that had migration or invasion were fixed and stained in a dye solution containing 0.1% crystal violet and 20% methanol or stained with DAPI. The cells were then counted and imaged under an Olympus DP27-2 microscope.

**Cell Apoptosis Assay**

*Caspase-3/7* activity generated in intact cells was assessed as previously described [7]. 3,000 cells were planted into a 96-well plate. After 48h incubation at 37°C, protocol of a *Caspase-Glo 3/7* assay kit (Promega) was performed according to the manufacturer’s instructions and then assayed with Fluoroskan Ascent FL (Thermo).

**Wound Healing Assay**

Cells were seeded in a 6-well plate at a high density (80%-90% confluence) and incubated with RPMI 1640 containing 10% FBS. After reaching a confluent monolayer, cells were treated with 10 μg/ml mitomycin C (M0503, Sigma) for 2 h in order to suppress proliferation. Scratching was done in different groups and examined under a microscope. Afterwards, an RPMI 1640 medium supplemented with 1% FBS was used to incubate the cells for 48 h. The cells were then photographed at time points of 0 h, 24 h, and 48 h.

**Recombination BMP3 and inhibitor treatment**

Recombination BMP3 treatment studies with or without inhibitors were performed in HCT-116 cells as previously described [8]. After incubation in serum-free medium for 24 h, recombinant human BMP3 (hBMP3, 113-BP/FC, R&D Systems, 100 ng/ml) was added to the dishes. Action periods differed from 0.5 h to 24 h, which enabled particular study. For inhibitor studies, cells were pretreated for 1 h with either drugs DMH1(S-7146, Selleck), SB431542 (S1067, Selleck), SB525334 (S1476, Selleck), or ML347 (S7148, Selleck). Subsequently, the cells were treated with or without 100 ng/ml hBMP3 for 1 h or 24 h. The inhibitor studies were also carried out in both HCT116 and WiDr cells with stable BMP3 expression.

**Microarray Analysis**

Total RNA was isolated from HCT116-BMP3 and HCT116-Mock cells using a RNeasy Micro Kit (Qiagen; German) according to the manufacturer’s instructions. DNA-free total RNA samples were processed for analysis using the Human HT-12v4 Expression BeadChip (Illumina, CA, USA) at Genergy Biotech (Shanghai) Co., Ltd. These arrays cover the entire human genome and include more than 47,000 gene transcripts. Three biological replicates were performed to analyze each sample. The Illumina GenomeStudio was used to extract data and perform hybridization quality control, with data analysis performed using an Illumina BeadStudio Application to find differently expressed genes.

**References**

1. Lai Jp, Chien Jr, Moser Dr, Staub Jk, Aderca I, Montoya Dp, Matthews Ta, Nagorney Dm, Cunningham Jm, Smith Di, et al. hSulf1 Sulfatase promotes apoptosis of hepatocellular cancer cells by decreasing heparin-binding growth factor signaling. Gastroenterology. 2004;1:231-48.

2. Mylonas I, Jeschke U, Shabani N, Kuhn C, Kriegel S, Kupka Ms, Friese K. Normal and malignant human endometrium express immunohistochemically estrogen receptor alpha (ER-alpha), estrogen receptor beta (ER-beta) and progesterone receptor (PR). Anticancer Res. 2005;3A:1679-86.

3. Mao J, Li X, Chen W, Xu B, Zhang H, Li H, Wang L, Jin X, Zhu J, Lin G, et al. Cell cycle-dependent subcellular distribution of ClC-3 in HeLa cells. Histochem Cell Biol. 2012;6:763-76.

4. Liu C, Li J, Xiang X, Guo L, Tu K, Liu Q, Shah Vh, Kang N. PDGF receptor-alpha promotes TGF-beta signaling in hepatic stellate cells via transcriptional and posttranscriptional regulation of TGF-beta receptors. Am J Physiol Gastrointest Liver Physiol. 2014;7:G749-59.

5. Zou H, Molina Jr, Harrington Jj, Osborn Nk, Klatt Kk, Romero Y, Burgart Lj, Ahlquist Da. Aberrant methylation of secreted frizzled-related protein genes in esophageal adenocarcinoma and Barrett's esophagus. Int J Cancer. 2005;4:584-91.

6. Harden Sv, Tokumaru Y, Westra Wh, Goodman S, Ahrendt Sa, Yang Sc, Sidransky D. Gene promoter hypermethylation in tumors and lymph nodes of stage I lung cancer patients. Clin Cancer Res. 2003;4:1370-75.

7. Deveraux Ql, Leo E, Stennicke Hr, Welsh K, Salvesen Gs, Reed Jc. Cleavage of human inhibitor of apoptosis protein XIAP results in fragments with distinct specificities for caspases. Embo J. 1999;19:5242-51.

8. Stewart A, Guan H, Yang K. BMP-3 promotes mesenchymal stem cell proliferation through the TGF-beta/activin signaling pathway. J Cell Physiol. 2010;3:658-66.
