# Supplementary figures and images for: BMP3 suppresses colon tumorigenesis via ActRIIB/SMAD2-dependent and TAK1/JNK signaling pathways
Source: J Exp Clin Cancer Res. 2019 Oct 28;38:428. doi: 10.1186/s13046-019-1435-1 (PMC6819484; doi:10.1186/s13046-019-1435-1)

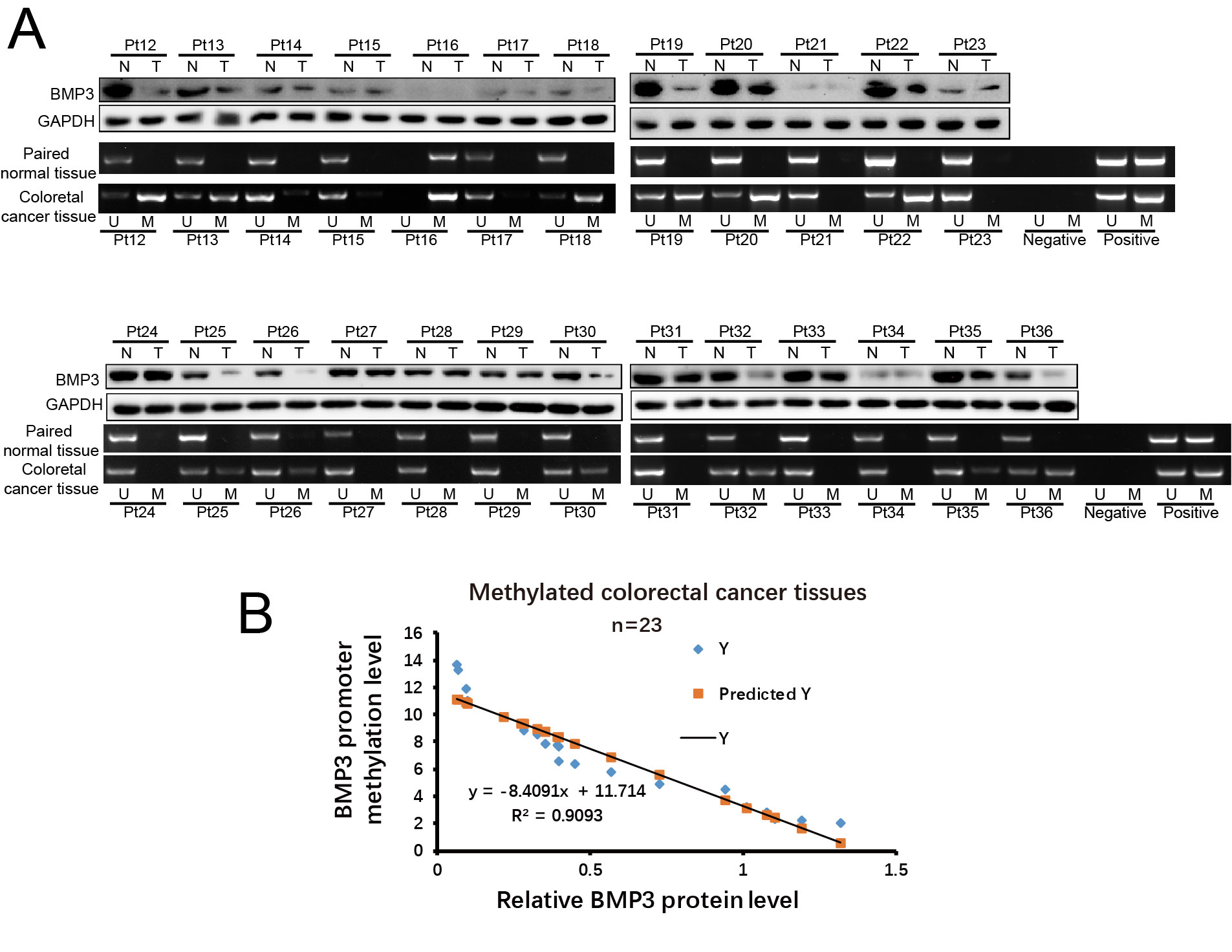

Supplement: Supplementary file 3 — Additional file 3: Figure S1. (A) Western blot and MSP analyses of BMP3 in additional CRC tissues and their paired normals (n = 25). N: Paired normal tissue, T: CRC tissue, U: Unmethylated, M: Methylated. (B) Correlation between the methylation level of BMP3 promoter and the relative BMP3 protein level of 23 methylated CRC samples by linear regression. Figure S2. (A) Cells were treated for 1 h (1 h) with or without 2 μmol/ml of DMH1, SB431542, SB525334, or ML347 in serum-free medium, followed with 100 ng/ml hBMP3 for another 1 h. Whole cell lysates were then subjected to western blot analysis using antibodies against p-SMAD2, p-TAK1, and p-JNK. (B) SB431542 was added to the cultured cells of HCT116-BMP3 and WiDr-BMP3 to detect the effects of the inhibitor on p-SMAD2, p-TAK1, and p-JNK by western blot. All experiments were repeated at least three times. Co: control. Figure S3. Geneontology (GO) enrichment analysis was performed in 3 categories (p < 0.05): (A) biological processes, (B) molecular functions, and (C) cellular components. Figure S4. Synchronized expression of caspase-7 and p21 in SCID mice xenograft tumors. BMP3 was stained brown in kytoplasm; caspase-7 was stained red in kytoplasm, and p21 was stained brown in nucleus. The scale bar is 40 μm. [file 13046_2019_1435_MOESM3_ESM.zip › Supplementary Figure S1.jpg]

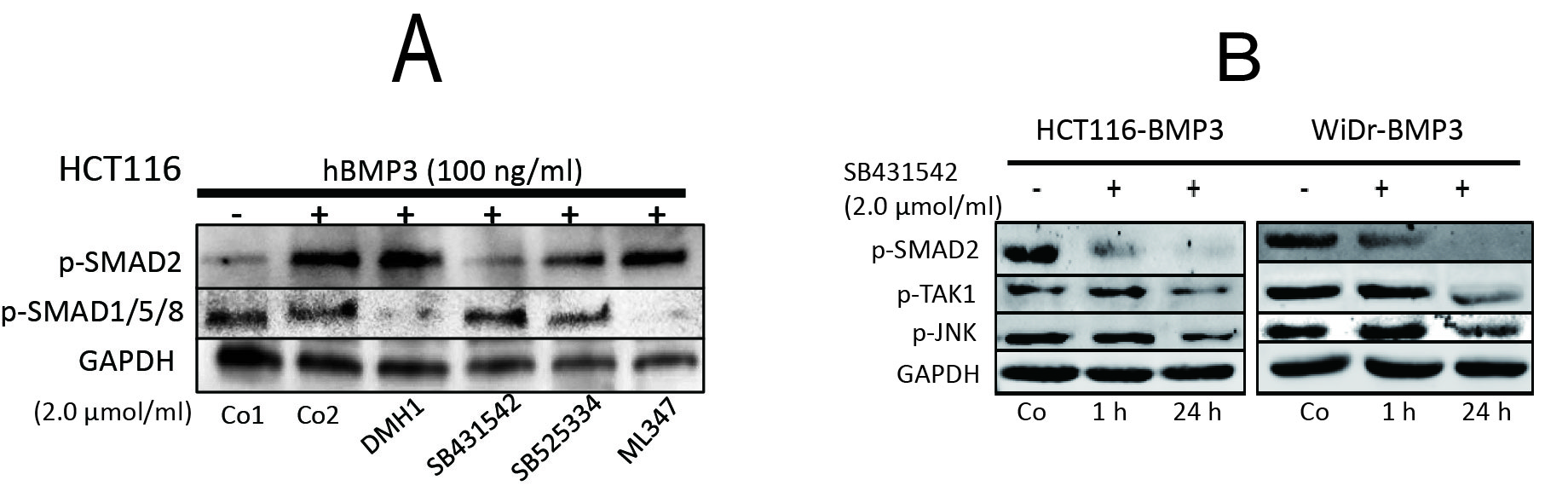

Supplement: Supplementary file 3 — Additional file 3: Figure S1. (A) Western blot and MSP analyses of BMP3 in additional CRC tissues and their paired normals (n = 25). N: Paired normal tissue, T: CRC tissue, U: Unmethylated, M: Methylated. (B) Correlation between the methylation level of BMP3 promoter and the relative BMP3 protein level of 23 methylated CRC samples by linear regression. Figure S2. (A) Cells were treated for 1 h (1 h) with or without 2 μmol/ml of DMH1, SB431542, SB525334, or ML347 in serum-free medium, followed with 100 ng/ml hBMP3 for another 1 h. Whole cell lysates were then subjected to western blot analysis using antibodies against p-SMAD2, p-TAK1, and p-JNK. (B) SB431542 was added to the cultured cells of HCT116-BMP3 and WiDr-BMP3 to detect the effects of the inhibitor on p-SMAD2, p-TAK1, and p-JNK by western blot. All experiments were repeated at least three times. Co: control. Figure S3. Geneontology (GO) enrichment analysis was performed in 3 categories (p < 0.05): (A) biological processes, (B) molecular functions, and (C) cellular components. Figure S4. Synchronized expression of caspase-7 and p21 in SCID mice xenograft tumors. BMP3 was stained brown in kytoplasm; caspase-7 was stained red in kytoplasm, and p21 was stained brown in nucleus. The scale bar is 40 μm. [file 13046_2019_1435_MOESM3_ESM.zip › Supplementary Figure S2.jpg]

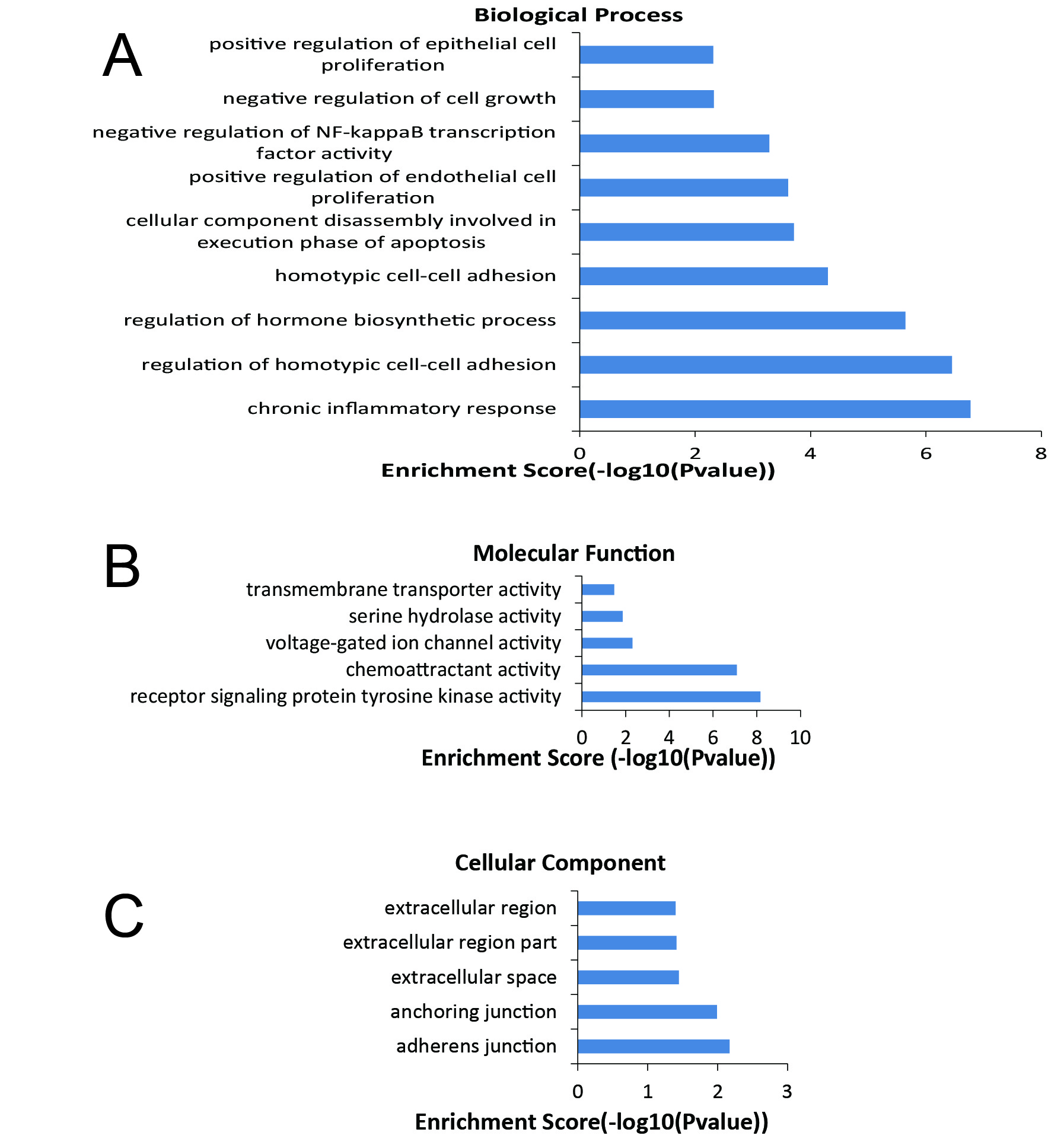

Supplement: Supplementary file 3 — Additional file 3: Figure S1. (A) Western blot and MSP analyses of BMP3 in additional CRC tissues and their paired normals (n = 25). N: Paired normal tissue, T: CRC tissue, U: Unmethylated, M: Methylated. (B) Correlation between the methylation level of BMP3 promoter and the relative BMP3 protein level of 23 methylated CRC samples by linear regression. Figure S2. (A) Cells were treated for 1 h (1 h) with or without 2 μmol/ml of DMH1, SB431542, SB525334, or ML347 in serum-free medium, followed with 100 ng/ml hBMP3 for another 1 h. Whole cell lysates were then subjected to western blot analysis using antibodies against p-SMAD2, p-TAK1, and p-JNK. (B) SB431542 was added to the cultured cells of HCT116-BMP3 and WiDr-BMP3 to detect the effects of the inhibitor on p-SMAD2, p-TAK1, and p-JNK by western blot. All experiments were repeated at least three times. Co: control. Figure S3. Geneontology (GO) enrichment analysis was performed in 3 categories (p < 0.05): (A) biological processes, (B) molecular functions, and (C) cellular components. Figure S4. Synchronized expression of caspase-7 and p21 in SCID mice xenograft tumors. BMP3 was stained brown in kytoplasm; caspase-7 was stained red in kytoplasm, and p21 was stained brown in nucleus. The scale bar is 40 μm. [file 13046_2019_1435_MOESM3_ESM.zip › Supplementary Figure S3.jpg]

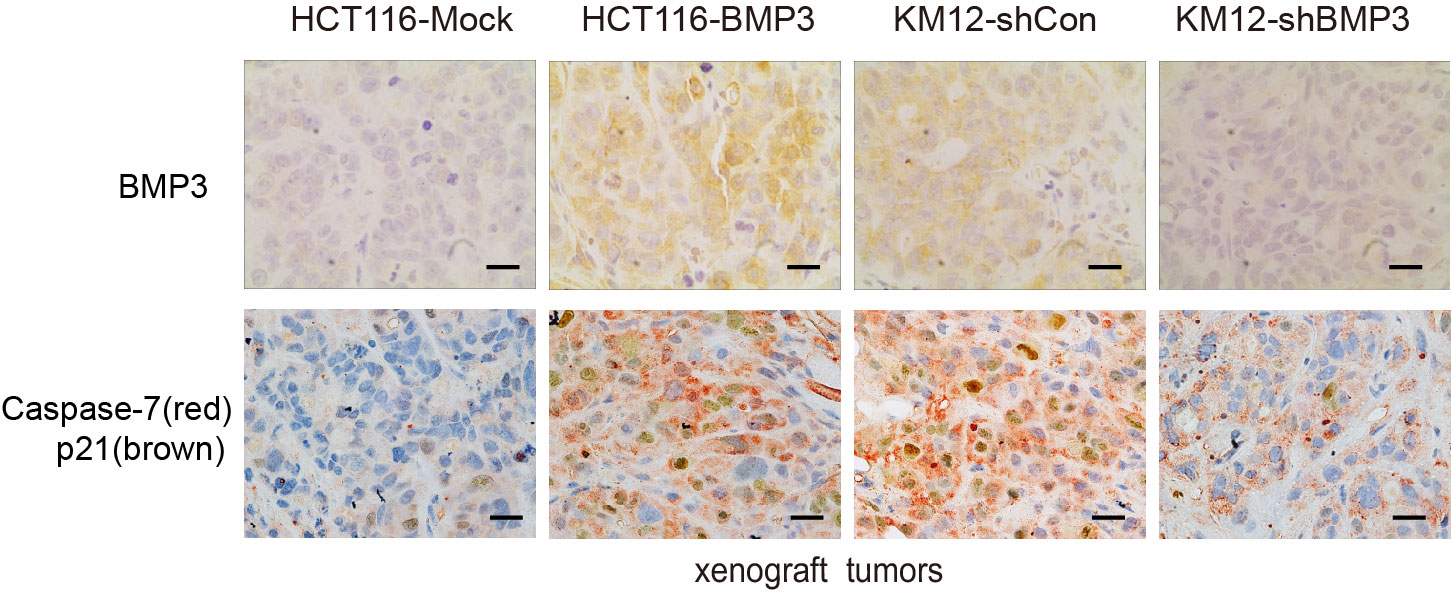

Supplement: Supplementary file 3 — Additional file 3: Figure S1. (A) Western blot and MSP analyses of BMP3 in additional CRC tissues and their paired normals (n = 25). N: Paired normal tissue, T: CRC tissue, U: Unmethylated, M: Methylated. (B) Correlation between the methylation level of BMP3 promoter and the relative BMP3 protein level of 23 methylated CRC samples by linear regression. Figure S2. (A) Cells were treated for 1 h (1 h) with or without 2 μmol/ml of DMH1, SB431542, SB525334, or ML347 in serum-free medium, followed with 100 ng/ml hBMP3 for another 1 h. Whole cell lysates were then subjected to western blot analysis using antibodies against p-SMAD2, p-TAK1, and p-JNK. (B) SB431542 was added to the cultured cells of HCT116-BMP3 and WiDr-BMP3 to detect the effects of the inhibitor on p-SMAD2, p-TAK1, and p-JNK by western blot. All experiments were repeated at least three times. Co: control. Figure S3. Geneontology (GO) enrichment analysis was performed in 3 categories (p < 0.05): (A) biological processes, (B) molecular functions, and (C) cellular components. Figure S4. Synchronized expression of caspase-7 and p21 in SCID mice xenograft tumors. BMP3 was stained brown in kytoplasm; caspase-7 was stained red in kytoplasm, and p21 was stained brown in nucleus. The scale bar is 40 μm. [file 13046_2019_1435_MOESM3_ESM.zip › Supplementary Figure S4.jpg]
