# Supplementary material for: BMP3 suppresses colon tumorigenesis via ActRIIB/SMAD2-dependent and TAK1/JNK signaling pathways
Source: J Exp Clin Cancer Res. 2019 Oct 28;38:428. doi: 10.1186/s13046-019-1435-1 (PMC6819484; doi:10.1186/s13046-019-1435-1)
Supplement: Supplementary file 4 — Additional file 4: Table S1. shRNA sequences and Primers for PCR. Table S2. Immunohistochemistry showed the expression of BMP3 in tissue samples. [file 13046_2019_1435_MOESM4_ESM.docx]

| **Supplementary table 1. shRNA sequences and Primers for PCR** | | | |
| --- | --- | --- | --- |
|  |  | **Forward primer** | **Reverse primer** |
| RT-PCR | BMP3 | CCCAAGTCCTTTGATGCCTA | TGGTACACAGCAAGGCTCAG |
|  | ID1 | GGTGAGCAAGGTGGGATTC | GACACAAGATGCGATCGTCC |
|  | ID3 | ATTCTTTGAATCCGCGGCTC | CGTTCACAGCCCGCTTAAAT |
|  | SMAD4 | CCTGCTCCTGAGTATTGGTGTTCC | CTCTCAATGGCTTCTGTCCTGTGG |
|  | CASP7 | GCTGACTTCCTCTTCGCCTATTCC | TGCCTGGCAACTCTGTCATTCAC |
|  | CDNK1A | GCTGAGCCGCGACTGTGATG | CCTCCAGTGGTGTCTCGGTGAC |
|  | BMP2 | TGACGAGGTCCTGAGCGAGTTC | TGAGTGCCTGCGATACAGGTCTAG |
|  | FST | CTGCTGCTGCTCTGCCAGTTC | CCTCCTTGCTCAGTTCGGTCTTG |
|  | ACVR2B | AGACACGGGAGTGCATCTACT | GCCTATCGTAGCAGTTGAAGTC |
|  | GAPDH | CATCACCATCTTCCAGGAGCG | TGACCTTGCCCACAGCCTTG |
| MSP | BMP3-U | TTTAGTGTTGGAGTGGAGATGGTGTTTG | AAACACAACCAAATACAACAAAAT  AACAA |
|  | BMP3-M | TTTAGCGTTGGAGTGGAGACGGCGTTC | CGCGACCGAATACAACGAAATAACGA |
| Q-MSP | BMP3-BS | TTTCGTTGTATTCGGTCGC | GCTACGAAACACTCCGAAA |
|  | ACTB-BS | TTTGTTTTTTTGATTAGGTGTTTAAGA | CACCAACCTCATAACCTTATC |
| Proble | BMP3-prb | 5'-HEX TTCGGGTTTCGTGCGTTTTCGTTTT-BHQ1-3' | |
|  | ACTB-prb | 5'-Cy5 AGTGTTGTGGGTGTAGGTATTAATATTG-BHQ2 3' | |
| shRNA | BMP3 | GGCCA AATCTCATCG AGATATCTCG AGATATCTCG ATGAGATTTG GCC | |
|  | SMAD2 | CACCGCCAGTTACTTACTCAGAACCTCAAGAGGGTTCTGAGTAAGTAACTGGC | |
|  | TAK1 | CACCGGACATTGCTTCTACAAATACTCAAGAGGTATTTGTAGAAGCAATGTCC | |
|  | BMPR2 | CACCGCTTGTGATGGAGTACTATCCTCAAGAGGGATAGTACTCCATCACAAGC | |
|  | ActRIIB | CACCGGGAGTGCATCTACTACAACGTCAAGAGCGTTGTAGTAGATGCACTCCC | |
|  | Control | CACCGCAAGCAAATAGCTTGCTTGATCAAGAGTCAAGCAAGCTATTTGCTTGC | |

| **Supplementary table 2. Immunohistochemistry showed the expression of BMP3 in tissue samples** | | | | | |
| --- | --- | --- | --- | --- | --- |
|  | No staining | Weak staining | Moderate staining | Strong staining | Total |
|  | <10% | 10%-30% | 30%-50% | >50% |  |
| Normal (n=31) | 0 | 4 (12.9%) | 8 (25.81%) | 19 (61.29%) | Neg (12.90%)  Pos (87.10%) |
| Adenoma (n=52) | 2 (3.85%) | 22 (42.30%) | 15 (28.85%) | 13 (25%) | Neg (46.15%)  Pos (53.85%) |
| Carcinoma (n=37) | 11 (29.73%) | 13 (35.14%) | 10 (27.02%) | 3 (8.11%) | Neg (64.86%)  Pos (35.14%) |
| Neg, negative (<30%), Pos, positive (≥30%) [1]. | | | | | |

1. Kodach Ll, Wiercinska E, de Miranda Nf, Bleuming Sa, Musler Ar, Peppelenbosch Mp, Dekker E, van den Brink Gr, van Noesel Cj, Morreau H, et al. The bone morphogenetic protein pathway is inactivated in the majority of sporadic colorectal cancers. Gastroenterology. 2008;5:1332-41.
